# Unlocking Trust in Community Health Systems: Lessons From the Lymphatic Filariasis Morbidity Management and Disability Prevention Pilot Project in Luangwa District, Zambia

**DOI:** 10.34172/ijhpm.2021.133

**Published:** 2021-09-20

**Authors:** Joseph M. Zulu, Patricia Maritim, Adam Silumbwe, Hikabasa Halwiindi, Patricia Mubita, George Sichone, Chileshe H. Mpandamabula, Frank Shamilimo, Charles Michelo

**Affiliations:** ^1^Department of Health Promotion and Education, School of Public Health, The University of Zambia, Lusaka, Zambia.; ^2^Department of Health Policy and Management, School of Public Health, The University of Zambia, Lusaka, Zambia.; ^3^Department of Community and Family Medicine, School of Public Health, The University of Zambia, Lusaka, Zambia.; ^4^Department of Environmental Health, School of Public Health, The University of Zambia, Lusaka, Zambia.; ^5^Participatory Research and Innovations Management (PRIM), Lusaka, Zambia.; ^6^Rigor Data Research, Consultancy Firm, Lusaka, Zambia.; ^7^Ministry of Health, Lusaka, Zambia.; ^8^Department of Epidemiology and Biostatistics, School of Public Health, The University of Zambia, Lusaka, Zambia.

**Keywords:** Trust, Community Health Systems, Hydrocele, Morbidity Management, Disability Prevention, Zambia

## Abstract

**Background:** Surgery for hydrocele is commonly promoted as part of morbidity management and disability prevention (MMDP) services for lymphatic filariasis (LF). However, uptake of these surgeries has been suboptimal owing to several community level barriers that have triggered mistrust in such services. This study aimed at documenting mechanisms of unlocking trust in community health systems (CHSs) in the context of a LF hydrocele management project that was implemented in Luangwa District, Zambia.

**Methods:** Qualitative data was collected through in-depth interviews (IDIs) and focus group discussions (FGDs) (n=45) in February 2020 in Luangwa District. Thirty-one IDIs were conducted with hydrocele patients, community health workers (CHWs), health workers, traditional leaders and traditional healers. Two FGDs were also conducted with CHWs who had been involved in project implementation with seven participants per group. Data was analyzed using a thematic analysis approach.

**Results:** The use of locally appropriate communication strategies, development of community driven referral systems, working with credible community intermediaries as well as strengthening health systems capacity through providing technical and logistical support enhanced trust in surgery for hydrocele and uptake of the surgeries.

**Conclusion:** Implementation of community led communication and referral systems as well as strengthening health services are vital in unlocking trust in health systems as such mechanisms trigger authentic partnerships, including mutual respect and recognition in the CHS. The mechanisms also enhance confidence in health services among community members.

## Background

**Key Messages**

** Implications for policy makers**

- Comprehensive mapping and inclusion of credible actors and structures in community health systems (CHSs) is essential to strengthening the credibility and implementation of strategies aimed at addressing neglected health challenges.
- CHSs strategies should take into account appropriate community-based health promotion channels if they are to be compatible with, and trusted by the community.
- Wide consultation among community actors is vital in developing locally appropriate communication messages and strategies to counter myths and misconceptions about hydrocele.

** Implications for the public**

Community health systems (CHSs) play a key role in shaping the uptake of interventions aimed at addressing neglected health challenges such as hydrocele for lymphatic filariasis (LF) in endemic regions. The CHSs are dynamic and consist of interactions between multiple stakeholders with diverse interests, agendas, values and beliefs. The diversity and complexity of these relationships may trigger rumors, fear and mistrust within communities regarding health programs especially those dealing with sensitive or neglected health issues such as morbidity management and disability prevention (MMDP) services for hydrocele. This study shows that the involvement of local actors in developing and implementing community health interventions has the potential of increasing trust in health systems and uptake of interventions.

Lymphatic filariasis (LF), a neglected tropical disease, accounts for 1.36 million disability adjusted life years globally.^1^ Long term infection is characterized by disease manifestations that include lymphoedema, elephantiasis and hydrocele^0.1^ An estimated 120 million people are affected by LF, while 25 million men are thought to have hydrocele globally,^1^ a condition which has been classified as a neglected male reproductive health condition.^2,3^ Morbidity management and disability prevention (MMDP) services targeted towards hydrocele and lymphoedema patients form the second pillar of the Global Program for the Elimination of Lymphatic Filariasis.^4,5^ The basic MMDP package includes treatment for LF infections and episodes of adenolymphangitis (acute attacks), management of lymphoedema to prevent acute attacks and disease progression to elephantiasis and surgery for hydrocele.^2,6,7^

Zambia is one of the 34 countries in the African region in which LF is endemic, with up to 12 million people at risk of infection. The overall prevalence of the circulating filarial antigen is 7.4%, and regional distribution ranges from 1% to 54% across the ten provinces.^8,9^ Efforts to combat disease transmission through mass drug distribution and provision of MMDP care to those with LF chronic manifestations have been ongoing since 2014.^10^ However, effective coverage and uptake of MMDP services has been suboptimal in most endemic countries, including Zambia.^11^ In order to improve access to MMDP services in an endemic district in Zambia, the University of Zambia School of Public Health working alongside the Ministry of Health launched a pilot project to explore how implementation and operations research can be used to support the delivery of quality services to LF patients.^12^ A baseline assessment prior to the pilot found that prevailing community beliefs and practices such as gender norms, play an essential role in the adoption of MMDP services.^11^

Several studies have found that many community level barriers affect uptake of these MMDP services even when they are offered for free.^3,13-15^ These barriers include myths and misconceptions about the negative effects of surgery for hydrocele, preference for traditional medicines, local beliefs about the causes of the hydrocele, stigma as well as non-compliance to recommended treatments.^3,13-15^ For example, one study from Sri Lanka, reported that MMDP services were avoided as patients feared public awareness of their condition.^13^ Further, uncertainty, worries, and fear about health interventions at community level may trigger insecurity and mistrust hence reducing the adoption of these MMDP services.^3,13-17^

Communities play an important role in the delivery of MMDP interventions through their involvement in drug distribution, awareness creation and demand generation. Many low- and middle-income countries have adopted different community engagement strategies to improve uptake of MMDP services.^7,18,19^ Some of the strategies include the involvement of community health workers (CHWs) in promoting MMDP for LF.^7,19^ In such programs, the CHWs are the key actors of the community health system (CHS) who act as intermediaries between communities and the health system.^20^ A CHS is defined as “the set of local actors, relationships, and processes engaged in producing, advocating for, and supporting health in communities and households outside of, but existing in relationship to, formal health structures.”^21^ The CHS comprises both hardware elements, such as drugs and technologies, as well as software elements such as values, relationships and trust between the different actors.^21,22^

The implementation of programs within CHS, including MMDP services is often challenging due to multiple stakeholder relationships, the complexity of CHS contexts including confidentiality issues, values, beliefs, and the fragmentation of community-based programs.^19,22-27^ These contextual issues tend to undermine trust by community members in health services.^22,28-31^ Trust influences health systems overall functioning and performance by shaping human behavior towards health institutions/interventions.^32,33^ Trust has been defined as “the optimistic acceptance of a vulnerable situation in which the trustor believes the trustee will care for the trustor’s interest.”^34^ In this study, trust refers to a situation where a trustor (person with hydrocele) believes that the trustee, namely CHWs, community leaders and health workers (CHSs actors) will provide appropriate health services (surgery for hydrocele/MMDP services) that will effectively respond to the trustor’s health challenges.

Promoting trust is a complex endeavour as trust is dependent upon many things. Factors that may influence trust include communication, respectful interactions, fairness, competence and also individual perceptions of social trust.^35-37^ Thus, CHS programs need to employ innovative approaches for unlocking trust in order to increase ‘social value,’ health systems performance and uptake of health services.^38,39^ Building trust is even more important in efforts to promote access to and utilisation of MMDP services, particularly surgery for hydrocele.^39^

While community level barriers to accessing MMDP services have been widely documented,^3,13-15^ studies that explore how to unlock trust in CHSs in order to promote uptake of hydrocele services are limited. This study aimed at contributing towards addressing this gap by examining mechanisms for unlocking trust in the CHS in a pilot project that sought to improve uptake of MMDP for LF services in Luangwa district, Zambia. It provides useful insights given the increased global interest and investment in CHS,^40^ owing to their potential to leverage different community resources in attaining universal health coverage.^40^

## Methods

###  Study Setting

This study took place in Luangwa District of Zambia, which is among the areas with the highest mean circulating filarial antigen prevalence rates (18.8%).^41^ As is the case in many other parts of the Zambia, the health system in Luangwa District consists of hospitals, health centers and health posts. Health posts, which make up the highest proportion of health facilities in Zambia, are located at the lowest levels of service delivery, the CHS.^20^ CHWs and community leaders are some of the main actors who play an important role in delivering health services in the CHS.^20^ The district has 16 health facilities and health posts, and two-hospitals where LF patients can access MMDP services.

###  The Luangwa MMDP Project 

In order to improve the delivery and utilization of MMDP services in Luangwa District, a community driven intervention was implemented between January 2019 and March 2020 to provide MMDP services to hydrocele patients. The intervention was developed and implemented by stakeholders from the Ministry of Health, the University of Zambia-School of Public Health, Participatory Research and Innovations Management Zambia (a research institute) and community leaders in the District. It was aligned to the Zambia Elimination of Neglected Tropical Diseases National Masterplan (2019-2023) strategic objective of delivering MMDP services to LF patients including provision of hydrocele management services in endemic districts as part of meeting disease elimination goals.^11^ In addition, the project also aimed at generating evidence to guide the formulation of the Zambia National MMDP strategy. Key intervention activities included offering hydrocele surgeries (hydrocelectomies), development of information education and communication (IEC) materials on hydrocele prevention and management, conducting community outreach activities and referral of hydrocele patients to Luangwa District Hospital and Katondwe Mission Hospital (the only facilities with capacity to conduct hydrocelectomy). The project also held a one-week training for CHWs and health workers on communication of key LF health messages, community engagement, case identification and management of hydrocele and lymphoedema.

At the time of project inception, records from the District Health Office showed that the district had only 80 documented hydrocele cases. However, the project conducted a mapping exercise using the CHWs when this project started, and 264 hydrocele cases were identified. The post implementation evaluation report of the project showed an increase in the levels of acceptability and adoption of hydrocele surgeries within the district during the implementation period. At baseline only 14.3% of hydrocele patients had undergone surgery compared to 40.1% at the end-line.^42^ Furthermore, majority of the patients had been visited by a CHW to discuss issues related to prevention or management of hydrocele (90.6%) in contrast to 50.2% when the program started.^42^ In addition, the proportion of patients who did not know the cause of their condition dropped from 80.7% pre-implementation to 23.3% at the time of completion while those who could correctly identify mosquitoes as the disease vector rose from 13.2% to 98.6% at end line.^42^

###  Study Design

 A qualitative study design was adopted with data collected through in-depth interviews (IDIs) and focus group discussions (FGDs). The use of qualitative approaches was considered the most appropriate in exploring community actors’ perspectives on the implementation of MMDP services in the district. Questions in the interview guides covered various themes including; barriers and facilitators to the implementation of services, coordination and partnerships amongst the different community actors, community participation in implementation processes and recommendations on improved service delivery and utilization. Data collection was conducted in February 2020.

###  Study Population and Sampling Strategy

 Participants were recruited through purposive sampling strategies targeting multiple community actors that included Ministry of Health officials, staff from health facilities providing hydrocele surgery, CHWs, traditional leaders, traditional healers and community members including hydrocele patients. In selecting the patients for the interviews, a mixture of different characteristics such as age, severity of disease and area of residence or zone were taken into consideration. We sampled at least 1 patient from each zone where the project was being implemented. This allowed the study to capture a broad range of perspectives on the implementation of MMDP services within the local CHS. All the eighteen CHWs that had received training and been involved in implementing project activities were included in the study.

 Thirty-one IDIs were conducted with four health workers one per zone, four traditional leaders one per zone, one traditional healer, eighteen patients and four CHWs. We selected two highest performing CHWs and two lowest performing CHWs (one per category from each chiefdom) to participate in the IDIs in order to learn in detail their experiences in referring patients for services and community-based case identification and management. Finally, two FGDs were conducted with the remaining CHWs with seven participants per group, one FGD per chiefdom. A total of 45 participants took part in both the FGDs and the IDIs as shown in Table.

**Table:** Demographic Characteristics of Study Participants

**Type of Respondent **	**Age Range **			**Gender **		**Total**
	**20-30**	**31-40**	**≥41 **	**Male **	**Female **	
Patients	6	6	6	18		18
CHWs	7	7	4	9	9	18
Health workers		2	2	2	2	4
Traditional leaders			4	4		4
Traditional healer			1	1		1

Abbreviation: CHWs, community health workers.

###  Data Collection Procedures

 In order to have an independent assessment of the implementation of the project, four research assistants who have had previous experience in programme evaluation using qualitative approaches and conducting neglected tropical diseases research at the University of Zambia, School of Public Health were recruited. These research assistants had not been engaged in any of the project activities and the underlying assumption was that the different community actors who had been engaged would be able to speak freely on their implementation experiences. Prior to the collection of data, the research assistants underwent a three-day training workshop to familiarize themselves with the project, participant recruitment procedures and data collection guides that they would be using. Technical support was provided by three members of the project implementing team (JMZ, GS and PM) during training and the process of data collection.

 Upon completion of the training, the research assistants recruited study participants and conducted the IDIs. Hydrocele patients living in the district had been identified and registered by CHWs in a patient database during the onset of the pilot program and had provided their consent to be involved in data collection processes aimed at monitoring program progress. This list was used to select potential patients alongside the contact information of the CHWs, health workers and other community actors which was on record for those who were regularly involved in project activities. Participants were invited to take part in the study either through telephone or face to face conversations. The interviews and FGDs lasted between 45 and 90 minutes and were recorded. Data collection was conducted in English and Nyanja, the local language, in the absence of the project implementing team. Once the IDIs were complete, two members from the project implementing team conducted FGDs with CHWs as a way of triangulating and validating the information collected by the research assistants.

###  Data Analysis

Audio files were transcribed verbatim and where necessary translations were conducted before analysis. Data was analyzed using a thematic analysis approach.^43^ Once interviews were transcribed, all the authors individually read the transcripts to create codes on mechanisms for unlocking trust in the CHS. The preliminary codes developed by the individual authors on trust mechanisms were then discussed, applied to a small number of IDIs, key informants and FGD transcripts, re-discussed in an iterative process to develop final codes and themes. This iterative process involved continuously moving between the themes on barriers to accessing surgeries and mechanism for unlocking mistrust and back to the data. To promote validity of the findings from the assessment as well as the enhance uptake of findings, the results were disseminated in a meeting conducted in with stakeholders in Luangwa on May 30, 2020. The stakeholders included CHWs, community leaders, District Health Management Team, policy-makers from the Ministry of Health and LF patients. The stakeholders pointed out that the pilot had generated evidence on effective strategies to deliver services that can improve the quality of life of patients and provided practical recommendations to guide planning and implementation processes at district level.

## Results

The post-implementation evaluation of the community driven MMDP intervention showed a significant increase in the levels of acceptability and adoption of hydrocele surgeries within the district during the implementation period. In this section, we present a summary of the key barriers regarding adoption of surgeries for hydrocele and the mechanisms facilitating unlocking of trust in CHS which subsequently increased the uptake of the MMDP services (Figure).

**Figure:**
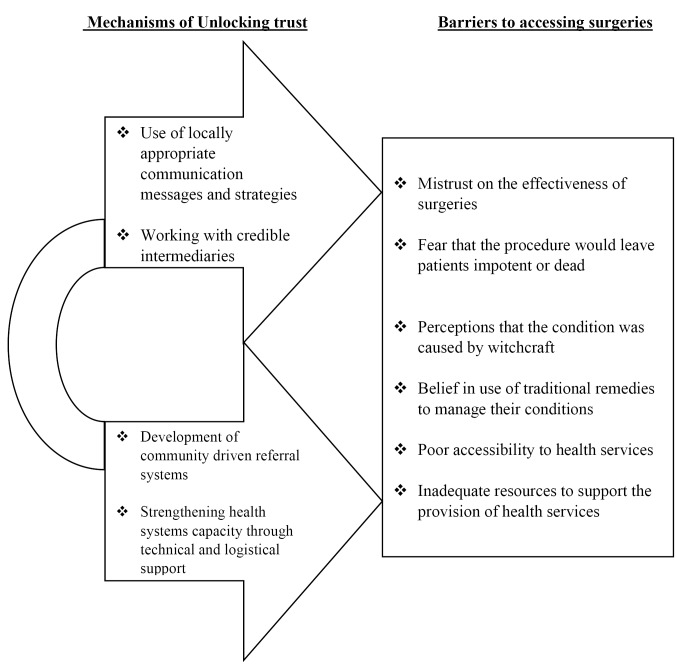
Key Community Barriers to Accessing Surgeries and Mechanisms Used to Unlock Trust.

 Several community level factors were found to have created a sense of mistrust on the effectiveness of surgeries in treating hydrocele thereby limiting access to hydrocele management services at the health facilities. These factors ranged from fear that the procedure would leave patients impotent or dead, to perceptions that the condition was caused by witchcraft and could only be healed through traditional medicine and practices. Despite all the hydrocele patients who were interviewed reporting regular use of traditional remedies to manage their conditions, they all stated that these remedies had not worked.

*“I tried going to traditional doctors, getting traditional medicines, I used to take the traditional medicines but I noticed that problem (hydrocele) was not getting healed” *(IDI 3, patient).

*“What made us not go to the health facilities is that we heard that if you go there for the operation, you would become impotent, and stop having children. So, we opted for traditional herbs, but then the problem was becoming worse” *(IDI 7, patient).

###  Mechanism of Unlocking Trust in Community Health Systems

#### Use of Locally Appropriate Communication Messages and Strategies

 In order to counter the misconceptions about the disease and available treatment options, the community-driven MMDP intervention delivered training to CHWs and health facility staff alongside behavior change campaigns targeted towards community members which were aimed at awareness raising and demand creation. CHWs and health workers who took part in the capacity building workshops on management of hydrocele reported feeling more confident in their ability to engage community members which heightened their motivation to conduct community sensitisation exercises.

*“The training equipped me with the information, and thus I was confident enough after being trained to challenge the myths and misconceptions about LF” *(FGD CHW 1, No. 2).

*“Previously, it was difficult to visit the people because we did not have enough knowledge, I always wondered what I would talk to the community about. But after being trained, I got motivated to visit the people, as I could talk about a lot of things such as prevention and management of hydrocele or lymphedema” *(FGD CHW 1, No. 8).

 Moreover, to develop culturally appropriate health promotion messages, a participatory approach was utilized whereby the key stakeholders reviewed and adapted existing materials to make them suitable for the community. Community stakeholders who were involved in the adaptation process included two chiefs from the district, ten traditional leaders, eighteen CHWs and health facility staff and officials from the Ministry of Health with support from the School of Public Health, University Zambia. The revised IEC materials which included booklets and posters were centered around five key health promotion messages focusing on disease transmission, prevention measures, who is affected by the disease, myths and management of hydrocele. These messages were piloted within community settings and feedback was used to refine the messages before translation into one of the local languages (Nyanja) so that community members could understand them with ease.

*“IEC materials made it easy for us to communicate our messages to community members including patients …, even us CHWs, we did not have adequate knowledge on LF, we learnt through the IEC materials that we received through the MMDP Project”* (FGD 2, No. 4).

 Additionally, the stakeholders also agreed to use pictures during community outreach activities as some of the people in the community were illiterate. The pictures depicted how hydrocele patients looked before and after successful surgery. Information on where to access the services and the necessary procedures was also provided. Interview participants pointed out that the use of pictures was an effective strategy as all the people in the community could easily understand them.

*“I was still in doubt as whether or not to access hydrocele services. But when I saw the pictures of how worse my condition might be if I did not access health services, I immediately decided to go for surgery” *(IDI 4, patient).

 The information was felt to have increased their trust in the health services compared to the traditional hydrocele management practices. The information was useful as the messages were deemed easy to understand and suitable for the community members as they addressed local values and beliefs about hydrocele.

*“Traditional beliefs and traditional doctors’ advice delayed me from accessing surgery. I also believed that the disease (hydrocele) comes from people’s magic and jealous. Fear of surgery made me not to access it. Some people in this village said that the doctor cuts the vein of reproduction during surgery, so I won’t have children, … and that I can also die. I believed in traditional medicines. But when this project came, with support from community health workers, I learnt that this disease is caused by mosquitoes- and can be cured by accessing health services like the one I accessed –the surgery” (*IDI 3, patient).

 During the implementation period, CHWs conducted regular sensitization meetings with community members and with hydrocele patients. The CHWs were provided with incentives such as lunch allowances when conducting outreach activities, branded T-shirts and bags which they stated had motivated them to conduct sensitization activities. This was because they felt that their work was being recognized and appreciated. Study participants felt that the sensitization meetings had resulted in enhanced knowledge and awareness of the causes, symptoms and management of hydrocele among the patients and other community members. One of patients provided an example on how their perception of the disease had shifted with the introduction of the intervention;

“*I heard that if you sleep with a woman during her monthly menstrual cycle, that is when the disease (hydrocele) comes. Then she (CHW) said this disease comes through mosquitoes and she taught very well. This teaching removed the doubt I had about hydrocele surgery” (IDI 17, patient).*”

 Despite conducting awareness raising on the importance of accessing services for hydrocele, myths and misconceptions regarding hydrocele surgeries still persist and have continued to affect access to health services. Fears that surgeries can cause death and infertility as well as disease related stigma still exist in the community especially among migrant and mobile fishing populations. The persistence of the fear and the stigma associated with the disease can be linked to the limited reach of project activities to these populations. Unlike static communities, who were easier to find as they were either in their homes or farms when CHWs were conducting outreach activities, mobile and migrant populations were harder to find and even when they were reached, they had fewer contact opportunities with the CHWs. As a result of stigma, other patients have opted to hide their condition due to fear of discrimination and ridicule. Both CHWs and hydrocele patients reported knowing someone with hydrocele who had not accessed services.

*“Some people still do not want to access surgeries. Some still fear that they may die especially fishermen who live far from health facilities. Some other people have fields in the neighboring country Mozambique and therefore shift and camp in that country for the period of the cultivation and harvest- making it difficult to reach them with information” *(IDI 16, patient).

*“Other patients have opted to hide their condition for fear of people stigmatizing them and this has led them to shy away from surgeries” *(IDI 30, health worker).

###  Development of Community Driven Referral Systems 

 The intervention facilitated development of CHW driven referral systems which included having referral forms managed by CHWs and setting up a patient database using information from door-to-door case identification carried out throughout the district. This had led to improved referral and communication processes between CHWs and health facilities. By promoting effective communication between community and health facility levels, once patients arrived at the facility they were attended quickly, enhancing their trust in the health services and increasing uptake of surgical services. This was important because during the formative phase of the intervention, hydrocele patients had pointed out that when they went to facilities, longer waiting times were a discouragement because of fear of possible social stigmatization or mixed messages from other people at the community.

*“The community health worker gave me a letter which I took to the nurse and doctor. Upon seeing that letter, they quickly attended to me without asking me questions. This was good because if you stay long in the queue or they ask you many questions, others may hear about your problem (hydrocele) and may laugh or stigmatize you” *(IDI 18, patient).

 The patient database also helped CHWs to carry out patient visits thereby strengthening follow-up systems. Some of them escorted patients for the surgical procedure or visited them at the health facility to see how the patients were recovering post operation.

*“For me, it was a situation of the client not coming back after a few days – and he had no phone to call the family. So, his parents came to me and said where is our child? So apart from the normal visit at his house, I had to go to the clinic to see how he was doing” *(FGD, CHW 1, No. 5).

 After discharge, CHWs made follow-up visits to ensure that the referral process was completed and a plan for the management of the wounds was being followed. On average, hydrocele patients stated that CHWs had visited them up to 4 times before and after surgery. However, depending on the severity of the case, the number of visits would increase. This was made easier by the fact that the CHWs knew where the patients resided and they could monitor the recovery process. In the FGDs, all the CHWs reported that they followed up their clients.

*“I visited him before and after the operation. Before surgery, the idea was to encourage him that all would be fine, and after surgery, I wanted to make sure that he was recovering well” *(FGD, CHW 1, No. 5).

*“I visited him once after he had done his surgery, but then when I went for second time to his house, I was told that he had gone to stay at his maize field as he wanted to focus on work, so I just decided to make calls” *(FGD, CHW 2, No. 8).

 Lack of transportation had also been identified as a logistical barrier to accessing services during the baseline assessment. To address this challenge, community actors had suggested that it would be important to provide the patients with a transport refund since most of them were very poor and could not afford the cost. Patients who were interviewed pointed out that the refund had been essential in mitigating this challenge.

*“They gave us money to use for transport, and for the operation and everything was free at the health facility. This motivated people to access services”* (IDI 1, patient).

 Nevertheless, in one of the hospitals providing surgeries, referring patients on the day of the operation did not give adequate time for the hospital to make the relevant plans for the surgeries. According to the hospital’s guidelines, patients are expected to arrive a day in advance so that they are examined, proper investigations carried out and then prepared for surgery.

*“We had a few cases where patients would come on the same day of conducting surgery. Now, this made us health workers to panic and change our program and start preparing for the surgery” *(IDI 29, health worker).

###  Working With Credible Intermediaries

 Engagement of the credible intermediaries such as traditional leaders, community members and traditional healers who were providing remedies for hydrocele or were hydrocele patients themselves had promoted uptake of hydrocele health services. One of the traditional leaders who was a patient and was using traditional remedies himself alongside other patients had undergone the surgery and as a result of the effectiveness of the treatment had decided to help others access the treatment too.

*“Am a traditional healer but I also failed. At last, I just decided, I needed to go for the operation for me to be okay. I came to know my medicines works for many other diseases – but not hydrocele. Looking at way the doctors worked, they worked very well, I do not have any complaints. The wounds were healed and things were removed” *(IDI 26, traditional healer).

*“As traditional leaders, we received information from the CHW. Then I accessed the services and I also teach people to go to the hospital and receive treatment, we have seen more people going there because they trust and respect us as their leaders. We have continued teaching this in our Tuesday village meetings” (IDI 25, traditional leader).*

 In another instance, a CHW engaged a traditional leader who lived with hydrocele for a long time but did not want to access health services. After being sensitized on hydrocele management using photographic IEC materials, the traditional leader agreed to access health services at the health facility. Upon being discharged, the traditional leader shared his experience with people in the community, a situation which made others also access the health services.

*“I knew a community leader, who was also a close friend and had hydrocele. I showed him the pictures on the stages of hydrocele and encouraged him to access the services. After accessing the services, he called a community meeting and told everyone about services, and that’s how many others also went for services” *(IDI 24, CHW).

 The CHWs who recorded the highest number of referrals were those that worked with the headmen and traditional healers in identifying and referring people with hydrocele to heath facilities. Being respected and recognized community stakeholders, engaging traditional healers and leaders made people also trust the health messages and services. For instance, one CHW reported that after successfully referring a traditional healer for hydrocele surgery, the traditional healer linked 10 of his hydrocele clients to the CHW for sensitization and referral to the health facility. All the ten patients successfully accessed the surgery services at the health facility.

*“After being trained, I talked to a traditional healer who was my relative and I knew had hydrocele and was treating others. I showed him the pictures of hydrocele and told him that if he did not address the situation, the condition will be worse. He told me that his wife had left him because of this problem. After undergoing surgery, he talked to his clients about me and the services at the health facilities. When the clients agreed to meet me, he gave a list of the people whom he was treating, and with his help we reached out to them, and they all accessed the services at the health facility” (IDI 23, CHW).*

 People were also motivated to access the services through seeing the benefits of undergoing an operation as they interacted with those who had accessed the services. These benefits ranged from reduction in pain to being able to work and do sports. Others also reported that there has been reduced stigma as a result of surgery.

*“Yes, they have benefitted because I can say those who were not coming out, feeling shy, when they saw that we are ok, they have started going there to access the services (hydrocele management)” *(IDI 11, patient).

 Patients who were married mentioned improved or better sexual relationships with their partners. One patient seemed to think or suggest that his wife was only able to be pregnant after had a surgery.

*“I encourage them to also go for surgery. I tell them that look at me, am just now okay and even my sexual performance at home is good, and it used to be a problem. Thanks to this the surgery, now my wife is pregnant” *(IDI 11, patient).

 Social support and encouragement from family members after seeing that others were getting better after accessing hydrocele management services also enhanced uptake of the services. In one case, a wife encouraged the husband to access surgery services based on what she had observed from people who had accessed the services.

*“My wife encouraged me to go for any operation. She said what people are saying about death or having no children is a lie. She said ‘look at that man from Musonda Village he was operated on and he never died*’” (IDI 5, patient).

 However, concerns that hydrocelectomy can result into death and infertility still exist especially in the fishing community. It was reported that such fears made a few people to opt out of an operation even after initially agreeing to do so. Programs supporting the delivery of these surgeries can integrate former patients who have successfully undergone the procedures in social and behaviour change campaigns and community outreach activities to help improve community acceptability.

“*One of the barriers that affected the implementation of the surgeries was the acceptability of the operation among hydrocele patients. Some patients were afraid of the operation because they thought that once they become anaesthetized for the operation, they would die” (IDI 30, health worker)*.

###  Strengthening Health Systems Capacity Through Technical and Logistical Support 

 During the formative phase of the implementation of the intervention, one of the main barriers that had been identified as impeding access to surgeries was the fact that a majority of the health facilities in the district did not have adequate resources to support the provision of the services. Consequently, one of the strategies employed as part of the intervention was providing financial support to the District Health Office to supply the facilities with materials and equipment needed for the surgeries. Improvement in the quality of services which was accomplished by ensuring that the services were readily available and delivered by trained and motivated health workers coupled with team work among the health workers reportedly increased community’s trust and confidence in the health services. Patients who expressed their satisfaction with the services they received to other community members motivated others to also seek surgeries for hydrocele.

*“What went well was the work competence of the doctors, the nurses and the community health workers, and also team work as these people worked together until the problem was solved” *(IDI 13, patient).

 Despite strengthening the referral pathways between the different health facilities to support the delivery of the surgeries, human resources for health and service delivery challenges were experienced. There are very few qualified health workers in the district and the surgeries are only provided in two district hospitals which meant that patients had to wait for some time on the list before they could be admitted at health facility. In one of the hospitals, health workers raised concerns about the sustainability of conducting the surgeries beyond the intervention’s lifetime. Hydrocele patients who were from lower economic groups would have to incur out of pocket costs because it was a Mission hospital and they are required to make payments to undergo the procedure.

*“This hospital usually incurs a charge for conducting the operations and keeping patients under observation before they can be discharged. This because they are not usually released on the day after the surgeries are conducted with the hospital stay lasting up to 48 hours after the surgery. The cost was being catered for by this project and with its completion, am not sure how the surgeries would continue being provided since most patients don’t have health insurance and they would have to pay for this cost out of pocket” *(IDI 29, health worker).

## Discussion

This paper reviews how the process of engaging CHWs, community leaders and health workers in designing and implementing a community driven intervention on promoting acceptability of hydrocele surgeries enhanced uptake of these surgeries. Involvement of community actors in designing and communicating health messages on hydrocele surgeries facilitated the development of information, which was locally appropriate. The messages were deemed suitable because community members found them simple and easy to understand and addressed local values, myths and beliefs about hydrocele. Data suggests that the simplistic and compatibility nature of the messages increased knowledge levels regarding hydrocele surgeries as well as appreciation and uptake of surgeries in the community. These findings support other studies that have shown that an intervention such as the community driven MMDP project is likely to be accepted if it has attributes that are perceived to be compatible with values and principles of the adopting systems or community; and if it is perceived as simple to use.^25,31^ A recent study in Zambia exploring uptake of community based interventions, emphasized the importance of taking into account local values and community structures when designing and communicating health messages as these may negatively impact people’s ability to participate in health interventions.^17^

The use of traditional healers, traditional leaders and patients who had successfully undergone surgery as community champions further promoted appropriateness and compatibility of the community driven MMDP intervention, and subsequently uptake of the surgeries. The CHWs who were able to make the highest numbers of patient referrals were those who, when in sensitizing the community on the importance of accessing surgeries, engaged traditional healers/leaders who had suffered hydrocele. This was because these community actors are viewed as credible intermediaries, and as such, their messages were often trusted by the community members. We therefore observe, like others, that identifying and engaging community members who truly represent the community in delivering health interventions may promote uptake of health services by increasing trust between health providers/ health system and communities.^17,44^ Use of locally appropriate channels and actors in the dissemination of health information has the potential for reducing mistrust in health systems by minimizing room for doubts and misinterpretation of health messages.^13,44^

Trust in healthcare systems and providers has been identified as an important social factor that influences health-promoting behaviors and utilization of health services.^42,44^ In this study, trust in the health system was further promoted through the provision of logistical and technical support. The support included training of CHWs and health providers on hydrocele management as well as health communication and community engagement, equipping health facilities with supplies and equipment and providing transport refunds for hydrocele patients. This finding is similar to other studies evaluating CHS which have shown that providing sufficient financial, human resources, supplies as well as knowledge and skills enhances trust in health interventions^45^ A recent paper on trust, CHWs and delivery of intermittent preventive treatment of malaria in pregnancy comprehensively documented that perceived ‘competence’ and ‘community and healthcare system integration’ underpinned communities’ trust in delivery of malaria services by CHWs.^46^ To promote sustainability of CHW driven activities, it is important to consider including standardized incentives and training for CHWs in the National Volunteer Policies. Further, it is also vital to prioritize equipping health facilities with enough supplies for undertaking surgeries and logistical support for hydrocele patients in the budget.

Uptake of services by the community was also promoted through utilization of community driven referral systems that involved CHWs in making patient referrals and accompanying them to health facilities. This system increased adoption of the services because it promoted ownership of the intervention at community level. The referral system also contributed to reducing stigmatization associated with hydrocele as well as the long waiting times. This is because the patients were attended to much quicker than usual. Further, the referral system also helped in strengthening relationships between persons with hydrocele and CHWs as they regularly visited the patients pre- and post-operation. Reduction in social stigma and stronger social relationships triggered trust and interest in the surgeries as the patients felt appreciated, respected and supported.^47^

 Our study demonstrates that leveraging the relationships and structures within communities is critical for successful implementation of interventions that are comprised of different components targeted towards diverse community actors. It also reflects the bidirectional nature of community processes, whereby the perceived trust in an intervention (such as use of CHWs and community leaders to deliver health messages and undertake referrals) and observable changes in community health outcomes, increases a community’s trust in the health system, which in turn increases their likelihood of adopting a specific intervention and health services in general. However, uptake of services among some community members such as migrant and mobile fishing populations still remains low, a situation which calls for further exploration of effective strategies for increasing the uptake of MMDP services among these populations.

 A limitation to the study is that two authors who were part of the implementation process of the MMDP for LF project participated in conducting a few interviews with CHWs. This may pose a potential bias in understanding and analysing findings as participants were likely to talk positively about the intervention to individuals who were evaluating/assessing ‘their own’ intervention – in this case the MMDP for LF project. To help mitigate this challenge and promote validity of the data, most of the data were collected and transcribed by people that were not involved in implementing the study. The rich description of phenomena, the study context, our multiple methods of data collection, diversity of respondents and use of quotes from different participants in the results section helped in developing an account what we believe provides a valuable contribution to the knowledge base on unlocking trust in CHSs. Further, the findings were disseminated and validated with stakeholders in Luangwa District during a dissemination workshop.

## Conclusion

 Engaging different community actors such as CHWs and traditional leaders in the design and communication of health messages on LF enhanced trust in and uptake of hydrocele management services as messages were locally appropriate. Community members expressed satisfaction with the messages as they were easy to understand and addressed local values and beliefs about hydrocele. The involvement of hydrocele patients, who had undergone surgery, in community sensitization further promoted trust and uptake of the surgeries as these actors were credible and respectable intermediaries. In addition, development of the community driven referral system triggered trust in and increased uptake of services by reducing stigmatization as well as strengthening social relationships between persons with hydrocele and CHWs. Finally, provision of logistical and technical support to health facilities also enhanced trust in hydrocele services. We recommend scaling up of delivery of locally appropriate communication messages and strategies among the migrant and mobile fishing populations in order to increase uptake of MMDP services among these populations. This will entail moving beyond the traditional community outreach and referral approaches that are designed around static community structures when designing service delivery approaches.

## Acknowledgements

 This work received financial support from the Coalition for Operational Research on Neglected Tropical Diseases (COR-NTD), which is funded at The Task Force for Global Health primarily by the Bill & Melinda Gates Foundation, by UK aid from the British government, and by the United States Agency for International Development through its Neglected Tropical Diseases Program.

## Ethical issues

 The study protocol obtained ethical clearance from the University of Zambia Research Ethics Committee (REF.017-11-18). Permission was also sought from the National Health Research Authority and relevant National as well as District authorities in Luangwa to conduct the study. The research team sought informed consent from all eligible participants before they could participate in the study. All participants provided written consent before taking part in the study. Voluntary participation of people in the community was ensued by emphasizing that participation in the study was not mandatory. Furthermore, the participants were assured that there would be no consequences if they refused to take part in the study, including loss of any health services. Confidentiality was observed throughout the study period by all those who were involved, with no disclosure of personal identifying information. All interviews were conducted in privacy either within health facilities or at household level to ensure confidentiality.

## Competing interests

 Authors declare that they have no competing interests.

## Authors’ contributions

 The study design was developed by all the authors. JMZ, GS, and PM participated in collecting the data. All authors participated performed the analysis, revised and approved the manuscript.

## Disclaimer

 The views expressed in the submitted article are those of the authors and not their funders

## Funding

This study was funded by the Coalition for Operational Research on Neglected Tropical Diseases (COR-NTD), (https://www.ntdsupport.org/cor-ntd/ntd-connector/term/ntdsc), under Grant Number:NTD-SC #163D and was awarded to CM.

## Authors’ affiliations

^1^Department of Health Promotion and Education, School of Public Health, The University of Zambia, Lusaka, Zambia. ^2^Department of Health Policy and Management, School of Public Health, The University of Zambia, Lusaka, Zambia. ^3^Department of Community and Family Medicine, School of Public Health, The University of Zambia, Lusaka, Zambia. ^4^Department of Environmental Health, School of Public Health, The University of Zambia, Lusaka, Zambia. ^5^Participatory Research and Innovations Management (PRIM), Lusaka, Zambia. ^6^Rigor Data Research, Consultancy Firm, Lusaka, Zambia. ^7^Ministry of Health, Lusaka, Zambia. ^8^Department of Epidemiology and Biostatistics, School of Public Health, The University of Zambia, Lusaka, Zambia.
